# Dr. Greeds, of Griefswald, on the Origin of Psychical Diseases

**Published:** 1863-01

**Authors:** 


### SELECTED.

### DR. GREEDS, OF GRIEFSWALD, ON THE ORIGIN OF PSYCHICAL DISEASES.

TRANSLATED FROM THE ALLGEMEINE ZEITUNG FOR PSYCHIATRIE, BY A. O. KELLOGG, M. D.

Up to the present time, men have been shy of attempting to interpret psychical processes upon physiological principles; yet it does not seem consistent to explain the mental activity by its analogy to other nerve-phenomena. For all life-activities (*labensthatigkeiten*) there is a structure in the central organ of the nervous system, which, as it were, stands bound to the peripheral parts of the body by guiding threads, partly to receive impressions from the outer world, and partly to interpose in the functions and emotions of the parts impressed. For motion, sensation, and mental activity, as well as for the vegetative functions, anatomical provision has already been pointed out; there are groups of cells, which, receiving impressions through the nerve-fibres, give forth their activities; distinctly formed cells have been found for individual functions (Shroeder von der Kolk :) not only the sensory are shown to be distinct from the motory by size and figure, but also, every sensory nerve has its particularly formed and regularly ordered ganglion-cells, from which it springs.

For the psychical functions, up to this time, we have not been able to find any distinctly marked anatomical paths. That such *exist*, however, we are forced to believe, and, indeed, it is not to be doubted, that the source of mental activity is in these, and that through them it is continually renewed and supplied.

Our knowledge of the minute structure of the brain extends to the roots of the sensory nerves. Why may we not be able to find in the remaining labyrinth of cells and connecting nerve fibres, which are either directly or indirectly in connection with the granules of the sensory nerves, the *laboratory of thought?* Already individual nerve-fibres are pointed out (Schroeder von der Kolk,) running between groups of cells, and which absolutely excite the peculiar activities of these. Wherefore should there not be also such conductors of the will for the separate cell-groups, which may serve as an anatomical basis for this or that circle of conceptions? Whether such will ever be demonstrated is indeed a question, but to accept the fact of their existence is, no doubt, justifiable. We shall be able to determine the anatomical traces of thought, when we shall have been able to find cells especially formed for the production of conceptions. That these lay on the outer surface of the brain, we are led to conclude from the fact, that disturbance of the regular course of thought is the usual result of inflammation of the membranes of the brain, and, in mental diseases, the stratum of cells on the outer surface of the hemispheres, as well as the ventricles, has been found degenerated.

The nervous activity in the new-born first manifests itself in the vegetative functions, motion, and feeling. Then the nerves of sense take up impressions, yet all is dark and unarranged, and the motions are to be regarded as phenomena of reflex action. Through these unconscious perceptions, through the operation of the outer world upon the organism, through the constant change of what is received, and the springing up of functions, there is gradually developed a distinct state of feeling, which has been designated as self-consciousness, common feeling, (*gemeingefhul.*) Upon the fortunate or unfortunate procedure of these functions, hangs, most significantly, the dispositions of men, which, by this dependence can be made very diversified. Indeed, from the manner, and according to the constant or changing development of these dispositions, the *temperaments* have been distinguished, which naturally are never the same in each individual man. With every self-conscious individual there is now formed, through physical dispositions, (desire and aversion,) and spiritual influences, (joy and sorrow,) a peculiar life-sensibility, (*gefuhlslaben*,) which we are commonly accustomed to denote as mind, soul, (*gemuth*.) Impressions are imparted to the brain through the nerves of sense, which reflect the condition of the outer world. Through repetition of such impressions, there is also created, without the co-operation of the organs of sense, but through the central activity, conceptions, which are diversified according to the diversities of the mind. Next, the child has only sensual conceptions. With the further development of the central organs, a higher order of ideas is formed,—ideas formed from the organs of sense, and the comparison of these, one with another. Most material is furnished by the organ of vision. This gives the conceptions of size, space, color, etc. The sense of hearing gives those of tone, noise, stillness, etc., and so every sense furnishes its distinct abstract ideas, which, by comparison and co-operation, furnish the complex operation of thinking. The faculty to operate with such thought-material is called *intellect*, understanding, (*verstand*.) Now, as the child passes by degrees from simple reflex movements to absolute capacities of emotion, there is also developed, as it were, out of the materials of conception, and under the influence of life-sensibility, (*gefuhlslaben*,) an organism for thought, which, by suitable education, can be perfected to a marvellous degree of fineness. This thought-organism now constitutes the spiritual being (*wasin*) of the man, his *soul* (*seele*,) his *I*, set free by the first approaches of self-consciousness at birth.

Glancing back at what has now been said, it is clear that the mental activity fashions (*auf baut*) the soul, and that the saying of Aristotle, "*nihil est in intellectu, quod non prius erat in sensu*," is undoubtedly true.

For an opposing or regulating force to the motory apparatus, there is a central function given, the will, which is also of influence in the excitation of pure central activity to the production of a succession of ideas; even as the will, by means of a single filament proceeding from the brain, can call into activity ganglion groups in the spinal cord, with the complex periphery of muscular nerves proceeding from it, so, apparently, can it call into activity groups of ganglion-cells in the brain, and excite them to the production of conceptions.

The similarity between these ganglion-cells and those of the electrical organs of certain fish, justifies the assumption that there is also a force generated in them, which calls forth the life-phenomena of the organism. If this apparatus is similarly charged, perhaps the condition may be designated as central expansion.

The signal of such central expansion is given, commonly, through the influence of the will; a more dark, unconscious sign of the same is designated as *impulse*, and with more complex functions as *instinct.* In vegetative life this unconscious discharge is a rudimentary principle.

Diseases of motility, as well as of sensibility, arise partly through faulty burdening of the central apparatus, and partly through obstructions in the regulating or conducting power, and thus we see, on the one side, spasm and paralysis, and on the other, pain and diversified disturbances of feeling. A similar condition is, indeed, apparent in mental diseases.

The causes of all mental diseases can doubtless be traced back to faulty burdening, or faults in the conducting or directing power of the nervous apparatus.

That the condition of the cerebro-spinal and vegetative systems are often co-operative, is shown by the circumstance that their roots generally run so near in connection that they cannot well escape common influences. Thus, some mental diseases begin with disturbances of feeling. The impressions of the outer world are either unperceived or falsely perceptible. The patient feels his members useless, or as if made of glass, or of wood; in his bowels he feels a creeping thing, muscular and common feeling is changed, and mental disturbances arise. These changed conditions of feeling are wont to precede melancholia, which, indeed, may not inappropriately be designated as cerebral paræsthesia. The abnormal sensations are falsely interpreted. The patient mistakes himself. Feelings of displeasure press heavily upon him; his will does not re-act, and we perceive with the approaching evil complete abulie, *melancholia attonita*, and catalepsy arising. I believe that all these conditions, with perhaps a healthy state of the conducting powers, may be traced back to defective burdening of the central apparatus. Melancholia contrasts strongly in every point with mania. Here we have elevated consciousness, happy disposition, irresistible and pressing conceptions, and muscular actions. The will no longer controls the impulsive explosion of the storms of emotion. In short, if the comparison will be allowed, a maniac gives the idea of a spark flying to a highly charged apparatus, lighting up, as it were, in it an involuntary discharge. Yet all these fluctuations of feeling, these anomalies of disposition, these pressing emotions, are not accustomed to continue long. The patient becomes composed, the effects disappear, he judges dispassionately, though often not less perversely, as to his condition. From this spiritual dejection (*gemuthskrankheit*) there results intellectual disease (*verstandeskrankheit :*) the secondary form is fashioned from the primary.

The melancholiac who has been driven to and fro by spiritual emotions, depends no more upon his former imaginations, loses by degrees all remembrance of them, forsakes forever his odd fancies, all their rule over his conceptions is lost, his thoughts no longer turn to the significance of abnormal sensations, self-consciousness disappears more and more, the old *I* crumbles under the storm of changed feelings, and there now only remains a planless entanglement of various kinds of spontaneous, self-engendered conceptions, without cohesion, and wholly undirected by the powers of the will; and from the melancholiac results the madman (*verruckter.*)

With raving madness (*tobsuchtingen*) things are fashioned quite differently. With elevated self-consciousness he believes himself foreordained, immensely rich, king, pope, etc. All his conceptions stand in relation to this circle of ideas; his augmented feelings of power deceive him as to the truth of this. So by degrees the old *I* is lost in the background, and from these fresh and complex conceptions, a new *I* is fashioned. The raving (*tobsuchtigen*) becomes the deluded (*wahn sinnigen*) maniac.

If the mental disease ends in complete weakness of all the bodily and mental functions, we have the form of dementia (*Blodsinn.*)

The course of insanity, when accompanied with rapid extinction of all nervous activity, furnishes the form known as paralysis.

Let us now seek a physiological explanation of the conditions just described.

It is an old and recognized physiological position, that the influences of the outer world aré only our own perceptions (sensations, *empfindungen.*) If now, with an incipient melancholiac, the intellectual centres perform their functions otherwise than they have been accustomed to—if he finds his limbs, or his intestines otherwise than what they have been—if he hears voices, or sees figures in the outer world which have no corresponding cause, he is led astry, and these come in contradiction with all his former experience. The explanation of these abnormal appearances falls upon another group of conceptions; the old will be neglected and a new series of cells set in motion, which the psychical act of explanation of these abnormal appearances interposes. The fluctuation in the state of the feelings causes likewise certain cell-groups to be called into requisition in such rapid alternation that the circle of cells which corresponds to them is particularly liable to be impinged upon by every trifling psychical irritation, and, like what we see in the motor department in St. Vitus' dance, the circle of conceptions is spasmodically and involuntarily loaded, so that the patient can no longer direct an ordinary course of thought by means of the will, and the spontaneous discharge from the group of conceptions is more or less given up. So by degrees every thing which capacity and education has previously built up in him is lost. The old paths, with cell-groups for distinct trains of ideas, obedient to the mandates of the will, become forsaken, obliterated as it were, and the spontaneous but irregular functions take new ones, and their activity furnishes anew the whole contents of the man. So that in place of a regular train of thought following the mandates of the will, there has arisen a dark, confused, spontaneous, self-engendered circle of conceptions.

If these new and faulty paths can once more be forsaken, and the authority of the will over the old group of conceptions be again established, then is the patient cured. On the other hand, if the old paths become impassable, an incurable madman is the image of this inner ideal disorder.

The equivalent of this in the motor department may perhaps be *paralysis agitans*.

If an incipient maniac, animated by feelings of the highest prosperity and inexhaustible power, is constrained to develope his might and accomplish great things, he soon becomes deluded as to his *personality*. The train of ideas which was accustomed to guide his own personality is lost, and a new path is struck out, which permits him to appear as King, Pope, Christ, etc. All imagination is drawn about this circle of ideas, and as his former personality was the result of early education, so the frequent impinging upon a new group of conceptions begets in him a new personality.

If now, there occurs in the course of the time, a tranquil rest to the heavy laden nervous apparatus, the group of cells which represents the old personality again comes into activity, and the patient is cured; but if these have now become impassable, then the complexity of cells which represents the new personality continue in function, and we have before us an incurable misconceited madman (*wahnsinnigen,*) who continually cultivates new and similar conceptions, that help to strengthen the new *I* (*Ich.*)

If, moreover, after the occurrence of irritation or inflammation, nothing transpires sufficient to arouse the cell-groups to renewed and healthy activity, and the patient becomes impassable, the power to form conceptions is either entirely lost, or becomes in the highest degree circumscribed. Commonly the cell-groups which correspond to the will are partially obliterated, so that ideal and emotional activity appear very imperfect. We have now before us the image of an imbecile (*blodsinnigen*—demented,) but one whose life, with sound vegetative activity, can long be preserved.

With a sudden breaking down of all cells affected by disease, we see the powers of emotion and conception rapidly vanish. This is the kind which usually suspends the nutritive changes in the peripheral organs, and precedes the rapid wasting away to speedy death. After conditions of exaltation, this is the usual end of the so much dreaded *paralysis.*

Now in conclusion, touching the development of the mental faculties in man, I believe that all the functions performed in the brain exist to this end, and only need education to be brought out. Even as the olivary, which may be regarded as an accessory ganglion of the hypoglossal granules, is far more remarkably developed in man than in animals, in whom the tongue performs very subordinate functions, even so we may suppose that with individual men, the granules of this or that sensory nerve, or the cells of this or that group of conceptions, may receive a correspondingly strong development. Education has now the task to discover the innate abundance or deficiency, and proceed according to this diagnosis to exercise more particularly the sparsely furnished cell-material, in comparison with that more abundantly bestowed in order to build up such a personality as may be developed from the material stock originally bestowed.

Every thing which excites the nervous system, or disturbs the circulation, furnishes a predisposition to mental disease. Whether this is brought about by psychical or mechanical causes, the effect is the same. It is by means of alterations in the preparation of the blood, and the irregularity of the nutritive changes in the brain-cells, that the anomalies of psychical functions are brought about.

That this is really the case, is shown by the transient condition of typhus, intoxication and narcotism, in which a transient insanity may be artificially engendered.

I will only remark, in conclusion, that those qualities of humanity, the divine nature of which can not be supposed to possess any inherent material, must remain comprehensively untouched by any physiological deductions.—*Jour. of Insanity.*

